# Sudden Death and Left Ventricular Involvement in Arrhythmogenic Cardiomyopathy

**DOI:** 10.1161/CIRCULATIONAHA.118.037230

**Published:** 2019-01-31

**Authors:** Chris Miles, Gherardo Finocchiaro, Michael Papadakis, Belinda Gray, Joseph Westaby, Bode Ensam, Joyee Basu, Gemma Parry-Williams, Efstathios Papatheodorou, Casey Paterson, Aneil Malhotra, Jan Lukas Robertus, James S. Ware, Stuart A. Cook, Angeliki Asimaki, Adam Witney, Irina Chis Ster, Maite Tome, Sanjay Sharma, Elijah R. Behr, Mary N. Sheppard

**Affiliations:** 1 Cardiology Clinical Academic Group, St George’s University Hospitals’ NHS Foundation Trust and Molecular and Clinical Sciences Institute, St George’s University of London, United Kingdom (C.M., G.F., M.P., B.G., J.W., B.E., J.B., G.P.-W., E.P. C.P., A.M., A.A., M.T., S.S., E.R.B., M.N.S.).; 2 Department of Pathology, Royal Brompton and Harefield NHS Foundation Trust, Imperial College London, United Kingdom (J.L.R.).; 3 National Heart and Lung Institute & MRC London Institute of Medical Sciences, Imperial College London, and Royal Brompton and Harefield NHS Foundation Trust, United Kingdom (J.S.W., S.A.C.).; 4 Institute of Infection and Immunity, St George’s University of London, United Kingdom (A.W., I.C.S.).

**Keywords:** arrhythmogenic right ventricular dysplasia, cardiomyopathies, death, sudden, cardiac

## Abstract

**Background:**

Arrhythmogenic cardiomyopathy (ACM) is an inherited heart muscle disorder characterized by myocardial fibrofatty replacement and an increased risk of sudden cardiac death (SCD). Originally described as a right ventricular disease, ACM is increasingly recognized as a biventricular entity. We evaluated pathological, genetic, and clinical associations in a large SCD cohort.

**Methods:**

We investigated 5205 consecutive cases of SCD referred to a national cardiac pathology center between 1994 and 2018. Hearts and tissue blocks were examined by expert cardiac pathologists. After comprehensive histological evaluation, 202 cases (4%) were diagnosed with ACM. Of these, 15 (7%) were diagnosed antemortem with dilated cardiomyopathy (n=8) or ACM (n=7). Previous symptoms, medical history, circumstances of death, and participation in competitive sport were recorded. Postmortem genetic testing was undertaken in 24 of 202 (12%). Rare genetic variants were classified according to American College of Medical Genetics and Genomics criteria.

**Results:**

Of 202 ACM decedents (35.4±13.2 years; 82% male), no previous cardiac symptoms were reported in 157 (78%). Forty-one decedents (41/202; 20%) had been participants in competitive sport. The adjusted odds of dying during physical exertion were higher in men than in women (odds ratio, 4.58; 95% CI, 1.54–13.68; *P*=0.006) and in competitive athletes in comparison with nonathletes (odds ratio, 16.62; 95% CI, 5.39–51.24; *P*<0.001). None of the decedents with an antemortem diagnosis of dilated cardiomyopathy fulfilled definite 2010 Task Force criteria. The macroscopic appearance of the heart was normal in 40 of 202 (20%) cases. There was left ventricular histopathologic involvement in 176 of 202 (87%). Isolated right ventricular disease was seen in 13%, isolated left ventricular disease in 17%, and biventricular involvement in 70%. Among whole hearts, the most common areas of fibrofatty infiltration were the left ventricular posterobasal (68%) and anterolateral walls (58%). Postmortem genetic testing yielded pathogenic variants in ACM-related genes in 6 of 24 (25%) decedents.

**Conclusions:**

SCD attributable to ACM affects men predominantly, most commonly occurring during exertion in athletic individuals in the absence of previous reported cardiac symptoms. Left ventricular involvement is observed in the vast majority of SCD cases diagnosed with ACM at autopsy. Current Task Force criteria may fail to diagnose biventricular ACM before death.

## Abstract 2

Supplemental Digital Content is available in the text.

**Clinical Perspective**

**What Is New?**

- In this large comprehensive autopsy study, we demonstrate that left ventricular involvement is observed in most decedents with arrhythmogenic cardiomyopathy and the left ventricle is exclusively involved in nearly a fifth of cases.
- Age at death, sex, normal macroscopic appearance of the heart, and participation in competitive sport were not associated with the presence of left ventricular involvement.
- We describe diagnostic histopathologic criteria for arrhythmogenic cardiomyopathy involving either or both ventricles.

**What Are the Clinical Implications?**

- This study identified that the heart was macroscopically normal in 20% of decedents with arrhythmogenic cardiomyopathy; expert pathological assessment, including histology, is therefore crucial to inform diagnosis in cases of initially unexplained sudden cardiac death.
- Left ventricular variants of arrhythmogenic cardiomyopathy may evade clinical detection using current diagnostic tools; this should be addressed in future revisions of Task Force criteria.

Arrhythmogenic cardiomyopathy (ACM) is a genetic heart muscle disorder characterized by myocardial atrophy and fibrofatty replacement of the ventricular myocardium. Originally described as arrhythmogenic right ventricular cardiomyopathy/dysplasia,^1,2^ increased recognition of left ventricular (LV) involvement has recently led to adoption of the term ACM.^3,4^ ACM has an estimated prevalence of 1:2000 to 1:5000 and is an important cause of sudden cardiac death (SCD) among young individuals, including athletes.^3,5,6^ ACM typically follows a Mendelian autosomal dominant inheritance pattern, where disease-causing mutations in >13 genes have been identified.^7^ Approximately half of the index cases with a clinical diagnosis harbor putative mutations in genes encoding desmosomal proteins: PKP2 (plakophilin-2), DSP (desmoplakin), DSC2 (desmocollin-2), JUP (junction plakoglobin), and DSG2 (desmoglein-2).

Clinical presentation of ACM is heterogeneous, and diagnosis can be challenging.^3^ This is reflected by the Task Force criteria, which integrate a number of structural, histopathologic, electrocardiographic, familial, arrhythmic, and genetic parameters.^6^ Moreover, these criteria are derived from cohorts with predominantly right ventricular involvement (arrhythmogenic right ventricular cardiomyopathy/dysplasia), and thus can potentially fall short for a significant proportion of those with LV dominant or biventricular disease. Task Force criteria require the presence of fibrofatty replacement of the right ventricular (RV) free wall myocardium on endomyocardial biopsy or at autopsy^6^; however, pathological and imaging studies have reported LV involvement ranging between 16% and 76% of cases.^8–12^ Cardiovascular magnetic resonance (CMR) may show late gadolinium enhancement, indicative of myocardial fibrosis, in a subepicardial or midmyocardial distribution, usually within the LV inferior or inferolateral walls.^13^

The purpose of this study was to report the pathological features of a large cohort of decedents that had experienced SCD from ACM, with a particular focus on the presence of LV involvement. In addition, we sought to report on the clinical characteristics and genetic associations of the cohort.

## Methods

The data, analytical methods, and study materials will not be made available to other researchers for purposes of reproducing the results or replicating the procedure. All unexpected sudden deaths in the United Kingdom are reported to the coroner, who determines the need for autopsy examination. The Cardiac Risk in the Young Center for Cardiac Pathology at St George’s, University of London, provides a nationally recognized expert cardiac pathology service. Referral is initiated voluntarily by the coroner’s pathologist after an unexplained SCD, or if there is suspicion of an inherited heart condition. The center receives ≈500 SCD cases per annum, just over half under the age of 35 years. A report to the coroner is issued within a 2-week period.

All referrals to the Cardiac Risk in the Young Center for Cardiac Pathology undergo a full coroner’s pathologist autopsy. Since 2013, spleen samples have been collected for postmortem genetic testing. Pathological findings and corresponding clinical information were entered into a database incorporating imaging from histological sections and autopsy/toxicology reports from UK coroners. Ethical approval for this study was obtained from the UK National Health Service Research Ethics Committee. Informed consent was provided by the next of kin at the time of referral.

### Study Group

**Figure 1.**
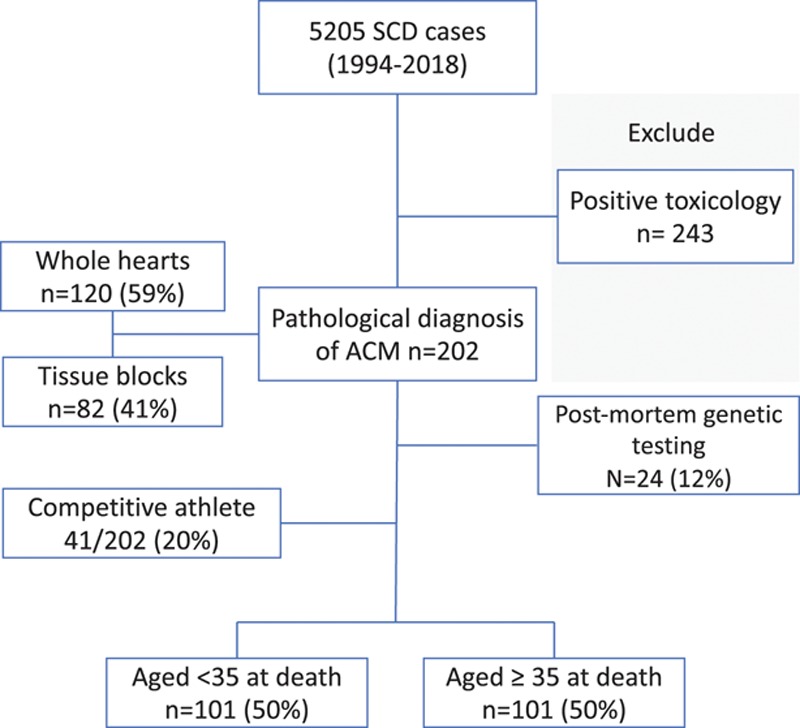
**Selection of ACM cases from all SCD referred to pathology center.** ACM indicates arrhythmogenic cardiomyopathy; and SCD, sudden cardiac death.

We evaluated 5205 consecutive SCD cases referred to the Cardiac Risk in the Young Center for Cardiac Pathology between 1994 and 2018 (Figure 1). Inclusion criteria for this study were: (1) SCD defined by one of the following: an unheralded witnessed instantaneous death, a death preceded by a prodrome of acute cardiac symptoms up to 1 hour before death, or an unwitnessed case without a previous deterioration in the preceding 24 hours; (2) pathological diagnosis of ACM at autopsy; (3) absence of an extracardiac cause of death; (4) negative toxicology screen; and (5) absence of obstructive coronary artery disease (atherosclerosis with residual luminal diameter <1mm^2^). Decedents with an antemortem diagnosis of cardiomyopathy were included.

### Demographics and Clinical Characteristics

A questionnaire was sent to all referring pathologists and coroners’ officers relating to demographics, medical history, symptoms, family history, sports participation, and circumstances of death. Additional information was obtained by autopsy reports from the referring pathologist, primary care/hospital correspondence, and family member interviews. This was acquired within 3 months of initial referral to our center. Media and sports club correspondence was also reviewed for competitive sports participation. Decedents were considered competitive athletes if they had engaged in an organized team or individual sport requiring regular training and participation in competitive events.^14^ For those with an antemortem diagnosis of cardiomyopathy, the clinical notes and investigations (including ECG, echocardiography, ambulatory cardiac monitoring, signal-averaged ECG, and CMR) were subsequently requested and analyzed according to the revised Task Force criteria.^6^

### Autopsy Evaluation

Whole hearts (3018/5205; 58%) and cases with tissue blocks (2187/5205; 42%) underwent detailed histopathologic analysis, including microscopic examination of tissue from both ventricles. Sections were fixed in formalin, embedded in paraffin, and stained with hematoxylin and eosin. The following areas were routinely examined: the right ventricular outflow tract; the anterior, lateral, and posterior right ventricle (midventricular level); interventricular septum; anterior, lateral and posterior left ventricle; the 3 major coronary arteries; and the ascending aorta. The apices of both ventricles were not examined in the absence of an overt structural abnormality. Information from the referring pathologist’s autopsy was also recorded, incorporating macroscopic appearances of the heart, body weight, and heart weight. At least 2 tissue blocks from both ventricles were required for study inclusion.

**Figure 2.**
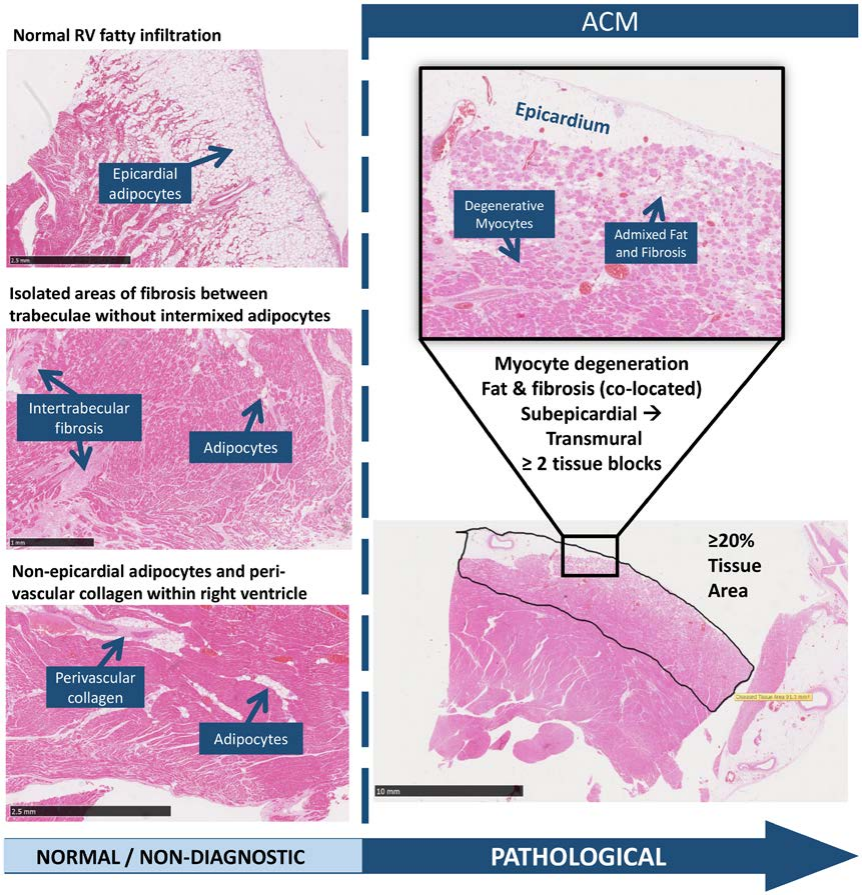
**Histological examination of ACM: normal findings within the RV vs pathological criteria for ACM.** ACM indicates arrhythmogenic cardiomyopathy; and RV, right ventricle.

ACM was defined on microscopic analysis as myocyte degeneration associated with intermixed fat and fibrosis (within the same microscopic field) from the subepicardial region inward or transmural in either or both ventricles (>20% in at least 2 tissue blocks of 4 cm^2^; hematoxylin and eosin stain). Disease involvement within the interventricular septum was defined by myocyte degeneration associated with intermixed fat and fibrosis from either side or within the midwall. This definition has been updated from a previous report.^15^ Figure 2 compares normal histological appearances in the right ventricle with a case meeting diagnostic criteria for ACM. All cases included in the study were examined by the senior author (M.N.S.), in addition to review by at least one other pathologist.

### Postmortem Genetic Testing

After extraction of DNA from retained splenic tissue (n=24), cardiac gene panel testing was performed using the Illumina TruSight Cardio (174 genes) panel (or a custom Agilent SureSelect with equivalent content; Table I in the online-only Data Supplement) and sequenced on the Illumina platform (NextSeq or HiSeq), as previously described and validated.^16,17^ Variant annotation was then undertaken in-house using SnpEff v4.3T (build 2017-11-24),^18^ GRCh37.75, and ANNOVAR (version 2017-07-17).^19^ Rare variants were defined as those with a minor allele frequency cutoff <0.01% in the Exome Aggregation Consortium general population database (http://exac.broadinstitute.org). Rare variants were then assessed for pathogenicity according to the American College of Medical Genetics and Genomics criteria.^20^ These criteria integrate a number of factors including population data, functional data, computational data, and segregation analysis to inform assessment of pathogenicity.

### Statistical Analysis

All variables were graphically inspected and summarized according to their nature and by outcomes of interest (ie, mean, SD, median) for continuous variables and proportions for binary or categorical variables. Data are presented as mean±1 SD or percentages and appropriate tests (*t* tests, [Fisher] χ^2^) were used for a preliminary flavor of variable associations.

There were 2 outcomes of primary interest, notably death occurring during physical exertion and LV histopathologic involvement. Univariable and multivariable logistic regression analyses were used to understand the strength of the crude and adjusted associations between these 2 outcomes and available demographics and clinical features in the ACM SCD group. The Hosmer-Lemeshow test was used to assess models’ goodness of fit. A *P* value of <0.05 was deemed statistically significant. Statistical analyses were performed using Stata IC/15 (StataCorp). The author (I.C.S.) holds responsibility for the statistical methodology applied to the data in this article.

## Results

### Baseline Demographics

Of 5205 cases of SCD referred to our unit, 202 (4%) were diagnosed with ACM. The majority of decedents from ACM were male (166/202; 82%) and white (182/202; 90%). The mean age of death was 35.4±13.2 years (median, 34.5 years).

### Clinical Characteristics

The great majority of cases (187/202; 93%) did not have an antemortem diagnosis of cardiac disease. Only 15 of 202 (7%) decedents carried a diagnosis of cardiomyopathy, of which 7 of 15 (47%) were labeled as ACM and 8 of 15 (53%) were labeled as dilated cardiomyopathy (DCM). Six of the overall SCD cohort with ACM (6/202; 3%) had an implantable cardioverter defibrillator in situ. Fifteen (7%) reported a family history of SCD under the age of 35 years, none of whom were diagnosed with cardiomyopathy in life. Two decedents with a positive family history had been referred for clinical investigation. Table 1 outlines clinical and pathological characteristics stratified by circumstances of death.

**Table 1.**
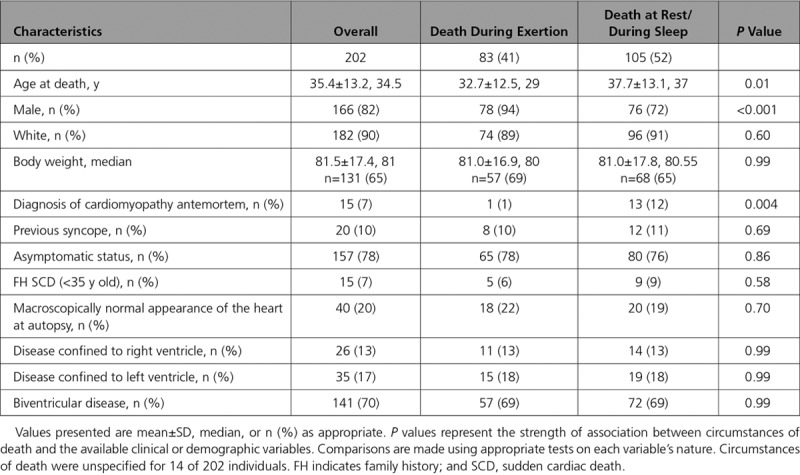
Clinical and Pathological Characteristics Stratified by Circumstances of Death

In most decedents with ACM (157/202; 78%), there were no reported symptoms before death; there were 20 (10%) instances of syncope recorded before death, which was associated with an antemortem diagnosis of cardiomyopathy in 6 cases. The remaining decedents (25/202; 12%) had a documented history of palpitations (8%), chest pain (2%), and presyncope (2%). Eighty-three (41%) of the overall cohort died during physical exertion, and 105 (52%) died at rest or during sleep. Decedents with an existing diagnosis of cardiomyopathy were more likely to have died at rest or during sleep in comparison with during physical exertion (87% versus 7%, respectively; *P*=0.004). Circumstances of death were unspecified in 14 of 202 (7%); there was no significant pattern in missing data with respect to other variables. Among young (<35 years old) decedents with ACM, there were more deaths during physical exertion than among older decedents (50% versus 33%, respectively; *P*=0.01).

### Pathological Characteristics

In the majority of cases (120/202; 59%), the whole heart was received for analysis. Of the remaining cases with tissue blocks (41%), the mean number of blocks received across both ventricles was 8.3±3.2 (range 4–15). In 40 of 202 (20%), macroscopic examination of the heart did not yield an overt structural abnormality (Figure I in the online-only Data Supplement). Heart weight and body weight were recorded in 193 of 202 (96%) and 131 of 202 (65%) cases, respectively, and demonstrated a positive correlation (*r*=0.55, *P*<0.001, n=129). Overall, 27% of men recorded heart weights >500 g, and 23% of women recorded heart weights >400g. The association between body weight and heart weight was independent of LV histopathologic involvement (n=129, *P*=0.74). After histological evaluation, none of the cases meeting diagnostic criteria for ACM showed myocardial inflammatory infiltrates.

### Ventricular Involvement

**Figure 3.**
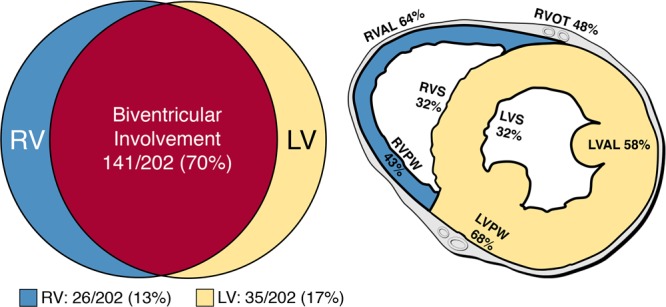
**Distribution and location of disease involvement in ACM. Left**, Ventricular disease involvement among all ACM decedents (n=202) **Right**, Distribution of fibrofatty infiltration among whole hearts referred to pathology center (n=120). ACM indicates arrhythmogenic cardiomyopathy; LV, left ventricle; LVAL, LV anterolateral wall; LVPW, LV posterior wall; LVS, LV septum; RV, right ventricle; RVAL, RV anterolateral wall; RVOT, RV outflow tract; RVPW, RV posterior wall; and RVS, RV septum.

An overview of ventricular histopathologic involvement is presented in Figure 3. LV fibrofatty infiltration was present in 176 of 202 (87%) decedents. Disease exclusive to the LV was observed in 35 of 202 (17%), and RV in 26 of 202 (13%), with 141 of 202 (70%) having evidence of biventricular disease. Among whole hearts referred to our center (120/202; 59%), the most common sites of LV disease involvement were the posterobasal wall (81/120; 68%) and anterolateral wall (69/120; 58%). In the RV, the anterolateral wall (77/120; 64%) and RV outflow tract (57/120; 48%) were most frequently implicated. A logistic regression model found no significant association between LV disease and age at death (*P*=0.31), male sex (*P*=0.58), macroscopically normal appearance of the heart (*P*=0.49), or competitive sport participation (*P*=0.27; Table II in the online-only Data Supplement).

### Competitive Athletes

Of 202 decedents with ACM, 41 (20%) had participated in competitive sport and the majority of the decedents (35/41; 85%) were aged <35 years. Thirty-one athletes (31/41; 76%) had been engaged in sports with a high dynamic component (Figure II in the online-only Data Supplement). The vast majority (37/41; 90%) of athletes died during physical exertion, including 30 athletes (30/41; 73%) who died during participation in sport. In contrast, 46 of 147 (31%) of the nonathlete decedents died during physical exertion (90% versus 31%, respectively; *P*<0.001). There was no significant difference in disease distribution within the heart between athletes and nonathletes (*P*=0.10).

Multivariable analysis showed that male sex (odds ratio, 4.58; 95% CI, 1.54–13.68; *P*=0.006) and participation in competitive sport (odds ratio, 16.62; 95% CI, 5.39–51.24; *P*<0.001) were independent predictors of death during physical exertion (Table 2).

**Table 2.**
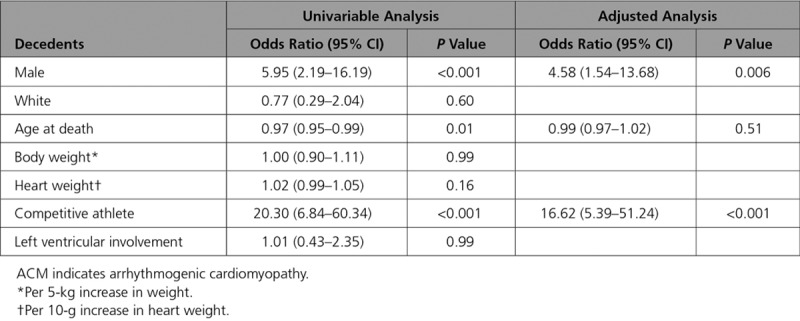
Analyses of Death During Physical Exertion Among ACM Decedents

### Genetic Testing

Genetic testing was performed in all cases with postmortem DNA available (n= 24 [12%]). A total of 27 rare variants were identified in 14 of 24 (58%) cases. Six decedents (6/24; 25%) hosted single pathogenic variants in known ACM genes: *DSP* (n=2; truncating mutations), *TMEM43* (n=2; missense mutations), and *PKP2* (n=2; frameshift mutations; Table 3). The same pathogenic *TMEM43* variant (p.Ser358Leu) was identified in 2 unrelated individuals and has been described in a number of different cohorts in the ClinVar database (https://www.ncbi.nlm.nih.gov/clinvar). None of the decedents hosted >1 pathogenic variant. Four decedents harbored rare variants of uncertain significance according to American College of Medical Genetics and Genomics criteria in desmosomal-related genes: 1 male with a pathogenic DSP variant hosted both missense and splice-site variants in *DSC2*; a previously reported rare missense variant was identified in DSC2 was identified in 1 decedent; and novel missense variants in *DSP* and *PKP* (variants of uncertain significance) were identified in 2 individuals also harboring pathogenic variants in these genes. All variants of uncertain significance are outlined in Table III in the online-only Data Supplement.

**Table 3.**
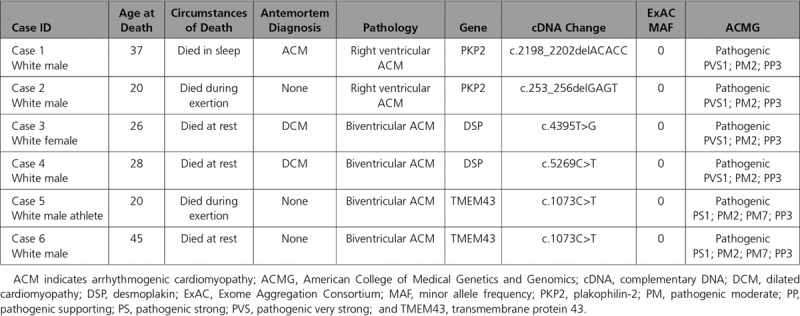
Overview of Pathogenic Variants Identified From Postmortem Genetic Testing

### Clinicopathological Correlations

**Figure 4.**
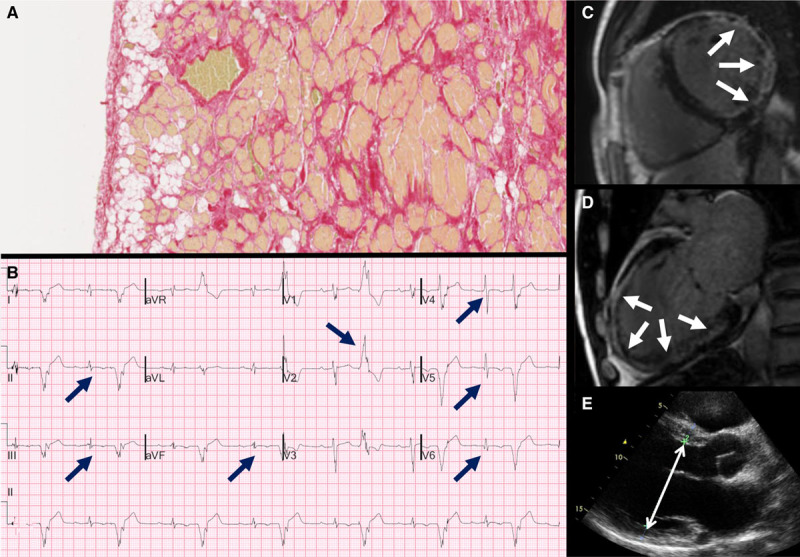
**A case of LV-dominant ACM. A**, Histological slide (Picrosirius red stain) demonstrating myocyte degeneration and fibrofatty infiltration within the posterolateral wall of the LV (extending transmurally). **B**, 12-lead ECG showing first-degree AV block, inferolateral T-wave inversion (arrows), and low-voltage limb lead QRS complexes, prolonged terminal activation duration in V_1_, and ventricular bigeminy with fragmented, broad, ectopics of RBBB morphology and superior axis (arrow). **C** and **D**, Delayed-enhancement CMR images illustrating extensive LV delayed enhancement, including near transmural enhancement of the lateral wall and midwall enhancement of the anterior wall. **E**, Parasternal long-axis view (echocardiography) showing severe LV dilatation (left ventricular end-diastolic dimension, 71 mm). ACM indicates arrhythmogenic cardiomyopathy; AV, atrioventricular; CMR, cardiovascular magnetic resonance; LV, left ventricle; and RBBB, right bundle branch block.

Clinical investigations were available in 10 of the 15 (67%) decedents with an antemortem diagnosis of cardiomyopathy. Seven subjects were diagnosed with DCM of which 4 had implantable cardioverter defibrillators in situ, and 3 decedents were diagnosed with ACM of which one had an implantable cardioverter defibrillator. Cardiac MRI had been performed in half and showed evidence of LV disease in 4 of 5 (80%). Modified Task Force criteria were applied retrospectively to all 10 cases. None of the decedents with a clinical diagnosis of DCM achieved a definite diagnosis of ACM by using Task Force criteria (Table 4). Figure 4 shows ECG, CMR, and echocardiogram appearances in an individual with LV-exclusive ACM diagnosed with DCM before death. Of 7 presumptive DCM diagnoses, none fulfilled major or minor Task Force criteria for imaging (Table IV in the online-only Data Supplement), whereas 6 (86%) exhibited T-wave inversion in at least one of the lateral leads (I, aVL, V_5_, and V_6_) and none demonstrated T-wave inversion in V_1_ through V_3_.

**Table 4.**
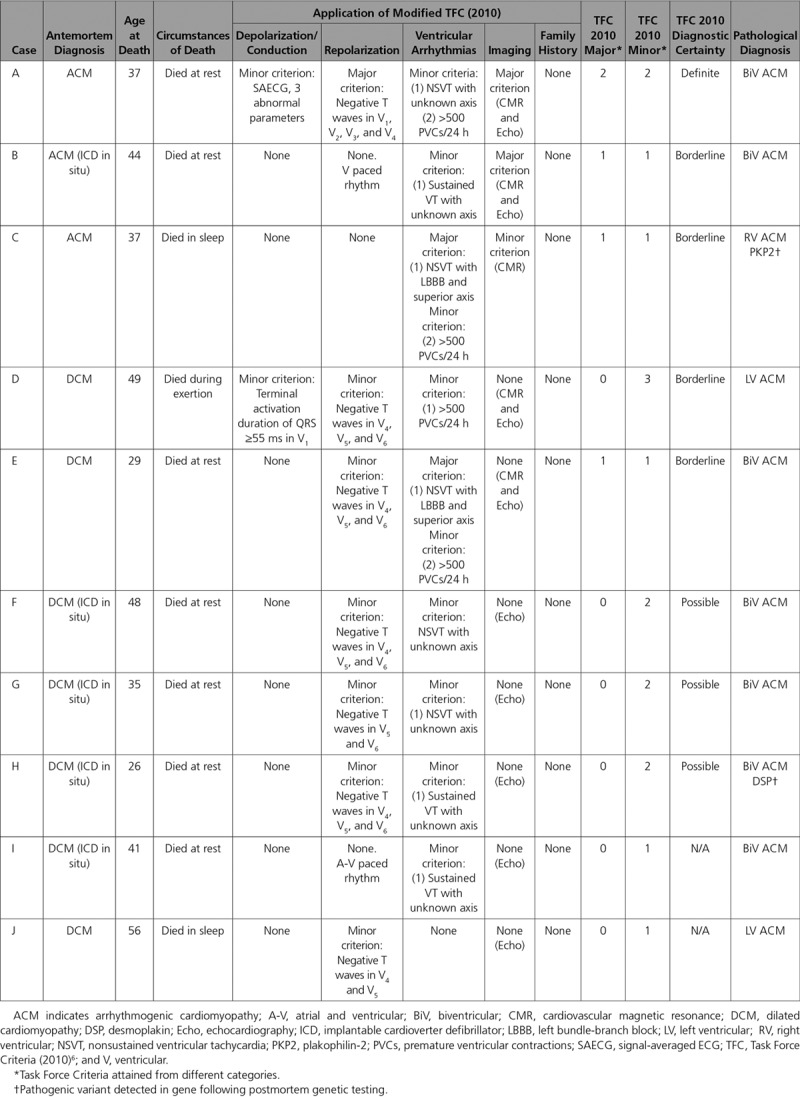
Clinicopathological Correlations Among ACM Decedents Diagnosed With Cardiomyopathy Antemortem

After autopsy examination, macroscopic appearances of the heart were abnormal in all 10 subjects. Postmortem genetic testing was undertaken in only a few and identified a DSP pathogenic variant in one DCM case, and a PKP2 pathogenic variant in an individual with ACM.

## Discussion

To our knowledge, we report the largest pathological series of ACM in SCD using contemporary definitions. We demonstrate that comprehensive histopathologic evaluation identifies LV involvement in the overwhelming majority (87%) of individuals experiencing SCD from ACM. This appears to be independent of age at death, sex, normal macroscopic appearance of the heart, and participation in competitive sport.

### Clinical Associations and Implications

Although ACM affects both sexes, men are more often phenotypically affected with a higher mortality rate and an increased incidence of cardiac arrest as the initial manifestation.^21,22^ These observations are consistent with our cohort, which was mainly male (82%). Furthermore, although death during physical exertion is well described,^15^ 52% of deaths in our study occurred at rest or during sleep. Syncope is also a common presenting symptom and marker of SCD risk. A quarter of a US cohort of patients with ACM (N=100) had a history of syncope,^23^ and an Italian registry of 301 patients with ACM reported that syncope was associated with a 3-fold increased risk of life-threatening arrhythmic events during follow-up.^24^ Nonetheless, most subjects in our study (78%) did not report any cardiac symptoms before presenting with SCD, and only 10% had experienced some form of syncope.

More than half of our decedents diagnosed with cardiomyopathy in life were labeled as DCM, but showed pathological features of ACM at autopsy. The overlap between DCM and ACM may present several diagnostic challenges as reflected in the finding that none of our decedents with an antemortem diagnosis of DCM fulfilled current Task Force criteria for a definite diagnosis of ACM. Among the antemortem DCM cohort, 6 (86%) exhibited T-wave inversion in at least one of the lateral leads: I, aVL, V_5_, and V_6_; none demonstrated T-wave inversion in V_1_ through V_3_. This highlights the phenotypic spectrum of ACM and the potential for biventricular disease to evade clinical diagnosis and subsequent risk management. We also identified a family history of young SCD (<35 years old) in 7% of the cohort, none of whom were diagnosed with cardiomyopathy during life. Guidelines recommend comprehensive family evaluation in this setting and may have led to the recognition of risk before death.^25^

### Myocardial Involvement at Autopsy

An early pathological study of 30 ACM hearts revealed LV involvement in 47% of their cohort.^8^ Patchy inflammatory infiltrates were also detected in 20 (67%) cases, a finding that was not replicated during histological examination of our study group. A later study of 42 cases showed macroscopic or microscopic features of LV disease in 76%, with a predilection for the septum and free wall (posteroseptal and posterolateral regions).^9^ In keeping with this and other previous studies, the posterobasal wall was the most common location of LV fibrofatty infiltration (68%) in our group. More recently, LV histological abnormalities were found in a lower proportion (32%) of 200 SCD cases attributed to ACM.^10^ However, fatty infiltration of the RV was considered pathological in this study, even in the absence of fibrosis. Our observations suggest that fatty infiltration of the RV should not be considered diagnostic for ACM. Our study also reports LV histopathologic involvement in a much higher proportion of decedents with ACM (87%). The role of inflammation in ACM has yet to be fully resolved. The lack of myocardial inflammatory infiltrates detected among our cohort is contrary to earlier assertions linking inflammation to the severity of outcome in ACM.^26^

### Correlation With Imaging Studies

The natural history of LV involvement in ACM is poorly understood. Our finding that LV involvement was not associated with age at death or macroscopic appearance of the heart at autopsy corroborates imaging studies that suggest LV involvement may occur at an early stage.^27^ Ghannudi et al^28^ evaluated 21 patients with ACM and found that 52% had LV involvement on CMR. The degree of RV impairment was similar between those with isolated RV involvement and disease affecting the LV. Rastegar and colleagues^29^ also studied 78 ACM mutation carriers of whom 38 had structural abnormalities on CMR, including the LV in 55%. The higher proportion of cases with LV involvement in our cohort may reflect a greater sensitivity of histopathology for fibrosis.

### Competitive Sport

The link between athletic activity and SCD risk in patients with ACM is widely acknowledged.^30^ A previous study found that participation in competitive sport was associated with a 2-fold increased risk of ventricular arrhythmia and SCD in ACM, but recreational sport followed a more benign course.^31^ Data from a prospective multinational registry of athletes with implantable cardioverter defibrillators also identified ACM as the only diagnosis associated with appropriate shocks during competitive sport.^32^ Current guidelines recommend against participation in competitive and endurance sport among individuals meeting Task Force criteria.^33^ Our data show that most competitive athletes who died with ACM were engaged in sports with a maximum dynamic component (ie, requiring the highest cardiac output), and most (90%) of these deaths occurred during physical exertion. We found no difference in either RV-exclusive disease or LV involvement between athletes and nonathletes, suggesting that exercise-induced remodeling alone is insufficient to lead to the ACM phenotype.

### Genetic Evaluation and Implications

To our knowledge, there have been no published studies evaluating the utility of postmortem genetic testing in ACM. Diagnostic yield may be influenced by several factors, such as disease presentation, family history, and geographic region.^34^ Our use of a stringent minor allele frequency threshold and application of American College of Medical Genetics and Genomics criteria may have resulted in a more conservative yield in comparison with previous genetic studies.^34,35^ Among decedents unheralded with cardiac disease, none had a reported family history of ACM. The only pathogenic variants identified in this group were in *TMEM43 and PKP2*, the same missense variant being identified in both (unrelated) *TMEM43* subjects. This variant had previously been reported in multiple ACM families internationally, is highly penetrant and has strong evidence for pathogenicity.^36^ Both probands exhibited histopathologic involvement of the LV, consistent with previous data on the mutation, which is associated with a particularly malignant form of ACM (Arrhythmogenic Right Ventricular Cardiomyopathy Type 5). *DSP* is also highly penetrant and associated with LV involvement and higher risk, especially truncating variants.^37,38^ Two subjects hosted truncating pathogenic *DSP* variants, and both had previous diagnoses of DCM.

### Implications for the Pathologist and Clinician

We demonstrated that 20% of ACM cases were macroscopically normal on initial inspection, regardless of LV involvement. Our findings are in line with earlier studies that recognize a concealed phase in ACM, where SCD may occur in the absence of overt structural abnormalities.^39^ This reinforces the importance of histological analysis of hearts in all cases of SCD. Moreover, previous reports have described sudden death occurring in PKP2 mutation carriers exhibiting structurally normal hearts at autopsy^16,40^; this may suggest a propensity for malignant arrhythmia in those with a pure electric phenotype.

In 17% of our cases, fibrofatty infiltration was identified exclusively within the LV, consistent with the previously described entity of LV-dominant ACM.^13^ LV-dominant ACM may manifest clinically with inferolateral T-wave inversion on the 12-lead ECG, arrhythmias of LV origin, and suggestive appearances on cardiac imaging. Thus, the absence of RV structural abnormality may lead to poor recognition of nonclassical disease utilizing current diagnostic criteria. Moreover, the significant minority of ACM SCD cases with LV-exclusive disease support inclusion of LV pathological and clinical markers in future revision of Task Force criteria.

### Study Limitations

This study relied on referral of SCD cases to an expert cardiac pathology center; therefore, there may be an element of referral bias because pathologists may choose to refer more challenging cases and potentially a higher proportion of cases with LV involvement. In 41% of cases, the whole heart was not sent to the center for analysis and macroscopic appearances of the heart were noted by the referring pathologist. Consequently, sampling technique may differ according to local protocols. However, tissue blocks from both ventricles were received in each case and histological diagnostic criteria consistently applied. Retrospective review of clinical information, competitive sports participation, and family member interviews could be subject to recall bias and may have underestimated the true extent of symptoms experienced in life that may otherwise have not been documented. Finally, although genetic testing was performed on all possible individuals with retained DNA for molecular autopsy, the group with genetic results is small, so the inference of genotype-mediated risk is inappropriate. A larger genetic testing uptake in the cohort would facilitate important genotype-phenotype correlations and assessment of genetic yield, which would be an important area worthy of future research.

### Conclusions

Most ACM-related SCDs occurred without preceding antemortem symptoms or relevant family history. Exercise-related SCD was observed in the majority of competitive athletes, with competitive sport participation conveying significantly increased odds of death during physical exertion. A fifth of cases demonstrated a macroscopically normal heart, emphasizing the importance of histopathologic evaluation in cardiac autopsy. LV involvement was present in the great majority of cases, with 17% exhibiting disease exclusive to the LV. Our study supports inclusion of LV structural involvement in future revision of Task Force diagnostic criteria.

## Acknowledgments

We are grateful to the charitable organization Cardiac Risk in the Young (CRY) for providing funding to support the CRY Center for Cardiac Pathology. We also thank the British Heart Foundation (BHF) for supporting the first author (Dr Miles) with a clinical research training fellowship (BHF Clinical Research Training Fellowship FS/18/28/33549).

## Sources of Funding

Dr Miles is funded by the British Heart Foundation (BHF Clinical Research Training Fellowship FS/18/28/33549), the Robert Lancaster Memorial Fund sponsored by McColl’s Retail Group Ltd, and Cardiac Risk in the Young (CRY). Drs Finocchiaro, Papadakis, Ensam, Basu, Parry-Williams, Papatheodorou, and Malhotra have received research fellowship grants from CRY. Dr Gray is the recipient of a National Health and Medical Research Council Early Career Fellowship (Fellowship 1122330). Drs Malhotra and Ware receive support from the National Institute for Health Research. Drs Behr and Ensam received research funding from the Robert Lancaster Memorial Fund sponsored by McColl’s Retail Group Ltd.

## Disclosures

None.

## Supplementary Material
