# Supplementary material for: Sudden Death and Left Ventricular Involvement in Arrhythmogenic Cardiomyopathy
Source: Circulation. 2019 Jan 31;139(15):1786–97. doi: 10.1161/CIRCULATIONAHA.118.037230 (PMC6467560; doi:10.1161/CIRCULATIONAHA.118.037230)
Supplement: Supplementary file 1 [file cir-139-1786-s001.pdf]

**SUPPLEMENTAL MATERIAL**

**Supplemental Table 1**. Gene List: Illumina TruSight Cardio (174 genes)

| | | | | | |
|---|---|---|---|---|---|
| ABCC9 | COL5A1 | GATAD1 | LAMP2 | PCSK9 | SMAD4 |
| ABCG5 | COL5A2 | GCKR | LDB3 | PDLIM3 | SNTA1 |
| ABCG8 | COX15 | GJA5 | LDLR | PKP2 | SOS1 |
| ACTA1 | CREB3L3 | GLA | LDLRAP1 | PLN | SREBF2 |
| ACTA2 | CRELD1 | GPD1L | LMF1 | PRDM16 | TAZ |
| ACTC1 | CRYAB | GPIHBP1 | LMNA | PRKAG2 | TBX20 |
| ACTN2 | CSRP3 | HADHA | LPL | PRKAR1A | TBX3 |
| AKAP9 | CTF1 | HCN4 | LTBP2 | PTPN11 | TBX5 |
| ALMS1 | DES | HFE | MAP2K1 | RAF1 | TCAP |
| ANK2 | DMD | HRAS | MAP2K2 | RANGRF | TGFB2 |
| ANKRD1 | DNAJC19 | HSPB8 | MIB1 | RBM20 | TGFB3 |
| APOA4 | DOLK | ILK | MURC | RYR1 | TGFBR1 |
| APOA5 | DPP6 | JAG1 | MYBPC3 | RYR2 | TGFBR2 |
| APOB | DSC2 | JPH2 | MYH11 | SALL4 | TMEM43 |
| APOC2 | DSG2 | JUP | MYH6 | SCN1B | TMPO |
| APOE | DSP | KCNA5 | MYH7 | SCN2B | TNNC1 |
| BAG3 | DTNA | KCND3 | MYL2 | SCN3B | TNNI3 |
| BRAF | EFEMP2 | KCNE1 | MYL3 | SCN4B | TNNT2 |
| CACNA1C | ELN | KCNE2 | MYLK | SCN5A | TPM1 |
| CACNA2D1 | EMD | KCNE3 | MYLK2 | SCO2 | TRDN |
| CACNB2 | EYA4 | KCNH2 | MYO6 | SDHA | TRIM63 |
| CALM1 | COL5A3 | KCNJ2 | MYOZ2 | SEPN1 | TRPM4 |
| CALR3 | COL5A4 | KCNJ5 | MYPN | SGCB | TTN |
| CASQ2 | COX16 | KCNJ8 | NEXN | SGCD | TTR |
| CAV3 | COL5A3 | KCNQ1 | NKX2-5 | SGCG | TXNRD2 |
| CBL | COL5A4 | KLF10 | NODAL | SHOC2 | VCL |
| CBS | COX16 | KRAS | NOTCH1 | SLC25A4 | ZBTB17 |
| CETP | CREB3L4 | LAMA2 | NPPA | SLC2A10 | ZHX3 |
| COL3A1 | COL5A3 | LAMA4 | NRAS | SMAD3 | ZIC3 |

**Supplemental Table 2**. Adjusted analysis of left ventricular involvement

| | Univariable Analysis | | Adjusted Analysis | |
|---|---|---|---|---|
| **Independent variable** | **Odds ratio (95% CI)** | **P value** | **Odds Ratio (95% CI)** | **P value** |
| Age at death | 1.03 (0.99-1.06) | 0.12 | 1.02 (0.98-1.06) | 0.31 |
| Male | 0.57 (0.16-2.00) | 0.38 | 0.70 (0.19-2.53) | 0.58 |
| Macroscopically normal heart | 1.39 (0.45-4.30) | 0.57 | 1.51 (0.48-4.74) | 0.49 |
| Competitive athlete | 0.52 (0.21-1.30) | 0.16 | 0.58 (0.22-1.53) | 0.27 |

CI indicates confidence interval.

**Supplemental Table 3**. Variants of Uncertain Significance (VUS) identified from post-mortem genetic testing

| ID | Age at death | Circumstances of death | Ante-mortem diagnosis | Pathology | Gene | cDNA change | ExAC MAF | CADD | ACMG |
|---|---|---|---|---|---|---|---|---|---|
| #100<br>Black Male | 39 | Died at rest | None | Biventricular | PRDM16 | c.481A>G | 0.0001 | 12 | VUS<br>PM2 |
| | | | | | DSC2 | c.1290G>C | 0.000008 | 19 | VUS<br>PM2 |
| | | | | | ABCG8 | c.287G>T | 0 | 17 | VUS<br>PM2; PP3 |
| #101<br>White Male | 53 | Died during exertion | None | Biventricular | CASQ2 | c.481A>G | 0 | 8 | VUS<br>PM2 |
| #102 (Case 6)<br>White Male | 45 | Died at rest | None | Biventricular | LDB3 | c.1444G>C | 0 | 32 | VUS<br>PM2; PP3 |
| | | | | | KCNH2 | c.755G>A | 0 | 2 | VUS<br>PM2 |
| #103<br>White Female | 34 | Died during exertion | None | Right ventricular | COX15 | c.520G>A | 0.00005 | 36 | VUS<br>PM2; PP3 |

| | | | | | | | | | |
|---|---|---|---|---|---|---|---|---|---|
| Athlete | | | | | | | | | |
| | | | | | LAMA2 | c.832G>T | 0 | 17 | VUS<br>PM2 |
| #104<br>White Male | 26 | Died at rest | None | Biventricular | RBM20 | c.2371C>T | 0 | 18 | VUS<br>PM2, PP3 |
| #105 (Case 1)<br>White Male | 37 | Died in sleep | ACM | Right ventricular | PKP2 | c.2197C>G | 0 | 14 | VUS<br>PM2 |
| | | | | | SCN5A | c.3916C>T | 0 | 12 | VUS<br>PM2 |
| #106<br>White Male<br>Athlete | 25 | Died during exertion | None | Biventricular | MYH11 | c.1528G>A | 0.00002 | 35 | VUS<br>PM2; PP3 |
| #107 (Case 4)<br>White Male | 28 | Died at rest | DCM | Biventricular | DSC2 | c.1918G>A | 0.000008 | 2 | VUS<br>PM2 |
| | | | | | DSC2 | c.354+3A>T | 0 | 13 | VUS<br>PM2 |

| | | | | | | | | | |
|---|---|---|---|---|---|---|---|---|---|
| #108 (Case 3)<br>White Female | 26 | Died at rest | DCM | Biventricular | DSP | c.1445G>A | 0 | 27 | VUS<br>PM2; PP3 |
| #109<br>White Male | 34 | Died at rest | None | Left ventricular | LAMA4 | c.1277T>C | 0.00009 | 18 | VUS<br>PM2 |
| #110<br>White Male | 23 | Died at rest | None | Biventricular | LAMA2 | c.8158G>C | 0 | 17 | VUS<br>PM2 |
| #111<br>White Female | 41 | Died at rest | None | Biventricular | FKTN | c.374G>T | 0.00003 | 16 | VUS<br>PM2 |

ACM: Arrhythmogenic cardiomyopathy; DCM: Dilated cardiomyopathy; cDNA: Complementary DNA; ExAC: Exome Aggregation Consortium; MAF: Minor allele frequency; ACMG: American College of Medical Genetics and Genomics; PM: Pathogenic moderate; PP: Pathogenic supporting; CADD: Combined annotation dependent depletion; VUS: Variant of uncertain significance.

**Supplemental Table 4**. Comparison of ante-mortem imaging with findings at expert post-mortem

| Case | Ante-mortem imaging findings | Macroscopic appearance of the heart | Heart weight | Pathological diagnosis | Microscopic description at cardiac autopsy |
|---|---|---|---|---|---|
| **A** | CMR: Normal sized LV with mildly impaired systolic function. Moderate-severe RV dilatation with moderate systolic impairment and akinetic free wall. Extensive LGE in RV free wall and trabeculae. Sub-epicardial LGE in LV lateral wall. | Dilated RV with fatty infiltration in the anterolateral and posterior wall. | 371 | Biventricular ACM | Transmural fibrofatty infiltration throughout the RVOT, anterolateral, and posterior RV wall. Fibrofatty infiltration within the LV posterolateral wall. |
| **B** | CMR: Non-dilated LV with normal systolic function. Hypokinesia of the basal anterior wall.<br>Dilated RV with severely impaired systolic function and free wall hypokinesia. LGE LV basal anterior wall. | Thin-walled, grossly dilated RV. | 490 | Biventricular ACM | Almost complete replacement throughout the anterior, lateral, and posterior wall of the RV by fat and fibrous tissue. On the right side of the IVS there is some admixed fat and fibrous tissue. There is involvement of the LV with fibrosis and fatty metaplasia in the outer one third, throughout the anterolateral and posterior wall of the LV. |
| **C** | CMR: Normal sized LV with preserved systolic function. Non-dilated RV with mildly impaired systolic function. RV free wall dyskinesia with wall thinning and microaneurysms. LGE base of RV and apex. | Fatty hypertrophy of RVOT and anterior wall of RV. Epicardial fat extending transmurally within the RV lateral wall. Posterior RV scar and wall thinning. | 522 | Right ventricular ACM<br><br>**PKP2 pathogenic variant** | Fibrofatty infiltration transmurally in the RVOT, anterior, lateral and posterior RV. Infiltration from epicardium into the sub-endocardium, also extending into the right side of the IVS in subendocardial fashion. There is no microscopic involvement of the LV. |
| **D** | CMR: Dilated LV with moderate impairment and akinesia of apex and lateral mid-wall. Moderate to severe eccentric LVH. Mild RV dilatation with low-normal RVEF. Extensive LV LGE, | LV dilatation and hypertrophy with scarring. | 644 | Left ventricular ACM | Epicardial LV fibrosis and fat with degenerate myocytes seen throughout (transmural fibrosis). |

| | | | | | |
|---|---|---|---|---|---|
| | almost transmural in lateral wall. | | | | |
| **E** | CMR: Dilated LV/RV with preserved biventricular systolic function. LV wall thinning, akinesia, and LGE within apical, anterior, inferior and lateral walls. | Epicardial fatty infiltration within the RV. IVS hypertrophy (18mm). | 484 | Biventricular ACM | Extensive fibrofatty infiltration anterior, lateral, and posterior RV wall. Circumferential fibrofatty infiltration within the LV. |
| **F** | Echo: Dilated LV with severely impaired systolic function. Inferolateral LV wall thin and akinetic. Non-dilated RV with normal systolic function. | Thinning of anterior, lateral, and posterior LV wall with scar in posterior wall and septum. | 425 | Biventricular ACM | Fibrofatty infiltration within the anterior and lateral RV wall. IVS: Mid-wall fibrosis. Fibrofatty infiltration within anterior, lateral, and posterior wall of the LV (outer third). |
| **G** | Echo: Mildly dilated LV with severely impaired systolic function. Non-dilated RV with good systolic function. RVOT free wall appeared hypokinetic. | Dilated thin-walled LV. | 490 | Biventricular ACM | Fibrofatty infiltration with degenerative features in the RVOT and RV anterior wall. In the mid-IVS there is fat and fibrous tissue with degenerative features, which extends onto the epicardial surface of the anterior, lateral, and posterior wall of the LV. |
| **H** | Echo: Dilated LV with moderate to severely impaired systolic function. Non-dilated RV with impaired free wall function. | Fat replacement in the RVOT, anterior, and lateral wall. Scarring is noted within left IVS and also circumferential LV (transmural in posterior wall). | 389 | Biventricular ACM<br><br>**DSP pathogenic variant** | Fibrofatty infiltration epicardial surface and transmurally in anterior, lateral, and posterior wall of the RV extending to the IVS, more on the right side. The changes extend over to the left to circumferentially involve the epicardial surface of the LV. |
| **I** | Echo: Dilated LV/RV with severely impaired biventricular systolic function. | Dilated LV with scar in the posterobasal wall. Fatty replacement of the RVOT, anterior, and lateral wall of the RV | 596 | Biventricular ACM | Fibrofatty replacement of the RVOT through the anterior and lateral wall, and partially in the posterior wall of the RV. Fat and fibrous tissue on the epicardial surface of the LV anterior wall but also the inner third involving trabeculae and this also applies to the lateral wall. In the LV posterior wall there is fat and fibrous tissue in the outer epicardium and inner subendocardial surface. |
| **J** | Echo: Dilated LV with severely impaired systolic function. Akinetic inferior wall. Non- | Dilated thin-walled LV. | 618 | Left ventricular ACM | Microscopic examination of the RV shows fatty infiltration of the outer wall but no fibrosis. In the anterior LV wall on the epicardial surface there is fat |

| | | | |
|---|---|---|---|
| | dilated RV with normal systolic function. | | and fibrous tissue replacing the outer third of the myocardium. This extends into the lateral wall and particularly the posterior wall. |

CMR: Cardiovascular Magnetic Resonance; Echo: Echocardiography; ACM: Arrhythmogenic cardiomyopathy; RV: Right ventricle; IVS: Interventricular septum; LV: Left ventricle; LVH: Left ventricular hypertrophy; LGE: Late gadolinium enhancement; RVOT: Right ventricular outflow tract; PKP2: Plakophilin-2; DSP: Desmoplakin; RVEF: Right ventricular ejection fraction.

**Supplemental Figure 1**. Normal macroscopic appearances of a heart with ACM

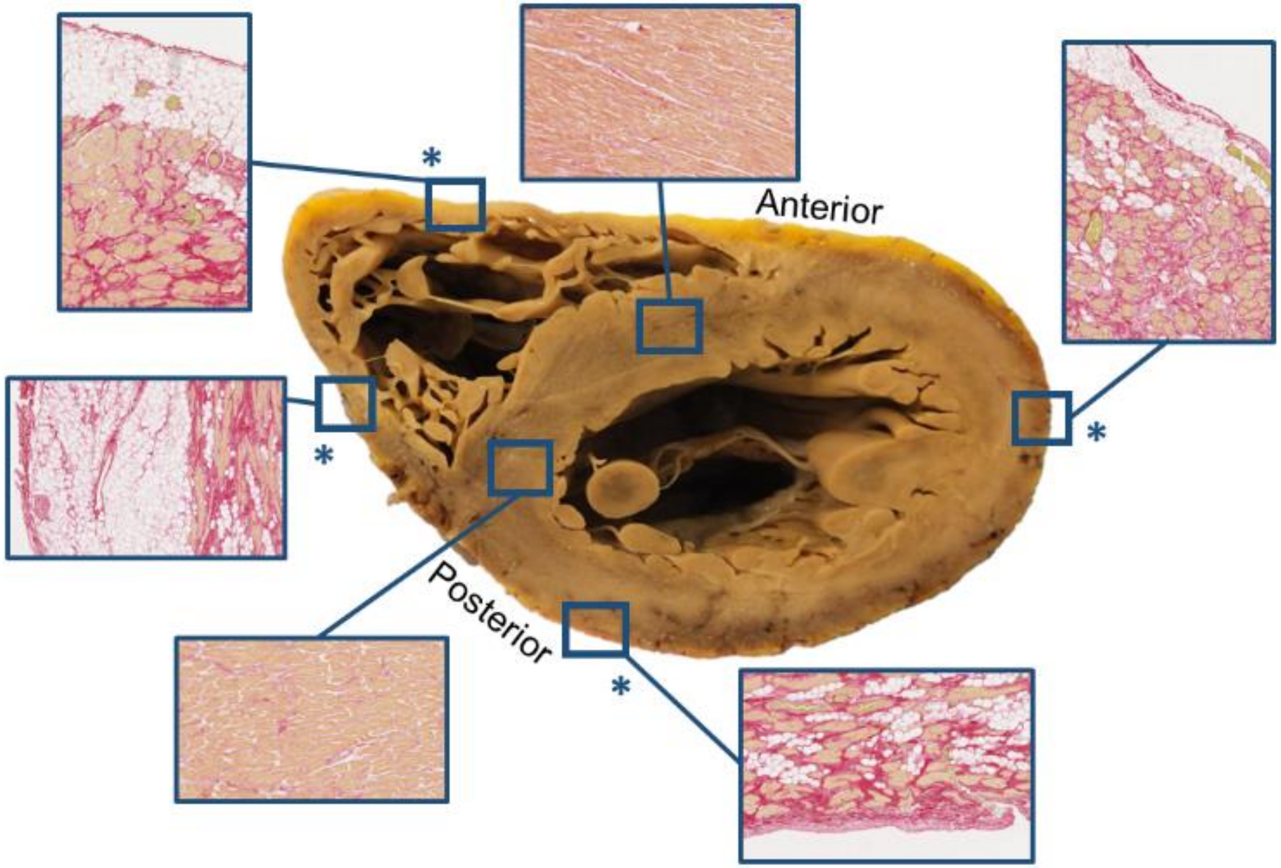


*Histological examination showed biventricular fibrofatty disease involvement within the anterior and posterior RV, anterolateral LV wall, and posterior LV.

**Supplemental Figure 2**. Classification of competitive sport according to static and dynamic component

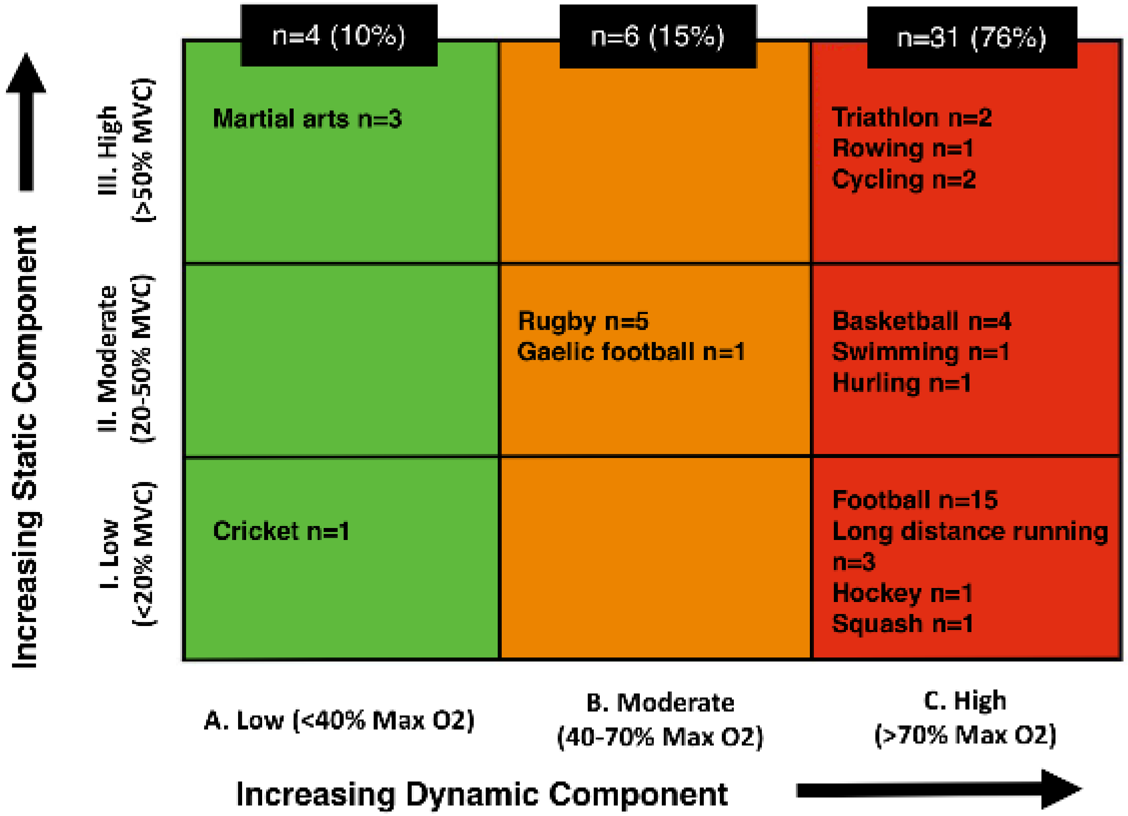


Modified from Mitchell et al. (1) with permission from Elsevier.

**Supplemental References**

(1) Mitchell JH, Haskell W, Snell P, Van Camp SP. Task Force 8: classification of sports. *J Am Coll Cardiol*. 2005; 45: 1364-1367.
